# Vertical ridge augmentation of atrophic posterior mandible with corticocancellous onlay symphysis graft versus sandwich technique: clinical and radiographic analysis

**DOI:** 10.1007/s10266-023-00794-0

**Published:** 2023-02-28

**Authors:** Yasser El-Sayed Fekry, Nermine Ramadan Mahmoud

**Affiliations:** 1 Lecturer of Oral and Maxillofacial Surgery, Oral and Maxillofacial Surgery Department, Faculty of Dentistry, October 6 University, Cairo, Egypt; 2 Associated Professor of Oral and Maxillofacial Surgery, Oral and Maxillofacial Surgery Department, Faculty of Dentistry, October 6 University, Cairo, Egypt

**Keywords:** Atrophic ridge, Posterior mandible, Ridge augmentation, Onlay graft, Chin graft, Sandwich technique

## Abstract

Alveolar ridge augmentation of atrophic posterior mandibular ridge represents a challenge in oral and maxillofacial surgery to restore aesthetic and function. The aim of the study was to compare the clinical and radiographic outcomes of bone formation in atrophic posterior mandibles augmented using onlay symphysis cortico-cancellous bone block with that augmented using sandwich bone augmentation technique (Inlay). Twelve patients were selected with missing mandibular posterior teeth. CBCT were done for all patients preoperatively to assess the residual bone height, ranged between 5 and 7 mm from the inferior alveolar nerve with adequate sufficient alveolar ridge width more than 4 mm. Patients required bone augmentation procedure with autologous onlay chin graft (group I) versus those used as inlay sandwich technique (group II). Clinical and radiographic analysis were done to analyses the newly formed bone and bone height. Percent of change in bone height was also calculated and revealed that group I was higher than group II, however, statistically insignificant differences between the two groups were found regarding the percentage of newly formed bone. Vertical ridge augmentation procedures using onlay chin graft took lesser time than the interpositional grafting with fixation technique, however, both techniques are promising for vertical ridge augmentation.

## Introduction

The lack of sufficient bone volume is one of the major challenges in dentistry. the inadequacy of alveolar ridge height or width requires alveolar ridge augmentation prior to implant placement [1]. Several bone grafting materials and techniques have been implemented to reconstruct the partially and totally edentulous aleolar ridges with relatively high rates of success. Those techniques include block onlay grafting, inlay grafting, guided bone regeneration using membranes with or without meshes, inferior alveolar nerve lateralization, inferior alveolar nerve transposition and distraction osteogenesis. Optimal technique selection depends on various factors including the magnitude of defect, grafted bone substitute material available, the medical status of patient and also the skill and surgeon’s experience [11, 14, 21].

The rationale of any grafting procedure was done to maximize the overlying graft blood supply aiming to prevent hypoxia which in turn resulted in ischemic changes at the distal portions of the flap which could eventually lead to wound dehiscence and graft failure [10, 24].

It has been stated that interpositionally grafting has the advantage of ensuring good vascularity to the graft, which should in turn result in lower resorption. There by the mobilized segment usually remains dimensionally stable because of the sustained periosteal blood supply, in addition to the graft endosteal incorporation from the adjacent bone marrow [8].

The aim of the study was to compare the clinical and radiographically outcomes of bone formation in atrophic posterior mandibles augmented using onlay symphysis cortico-cancellous bone block with that used as inlays sandwich technique.

## Materials and methods

Twelve patients (7 females and 5 males) with mandibular posterior atrophic ridge were selected from the Oral and Maxillofacial Surgery department, Faculty of Dentistry, October 6 University with age range between 24 and 40 years.

Inclusion criteria: bone height in the defect area ranged between 5 and 7 mm, measuring from the crest of alveolar ridge to inferior alveolar nerve, which was firstly measured by CBCT.

Exclusion criteria:

- Patients suffering from any systemic diseases were excluded from the study.
- Poor oral hygiene and motivation,
- General contraindications to implant surgery,
- Uncontrolled diabetes,
- Irradiation, chemotherapy, or immunosuppressive therapy over the past 5 years,
- Active periodontitis,
- Psychiatric problems

The patients were divided into two groups, six patients in each group. In the first group (I), bone augmentations procedure with autologous onlay chin graft versus (group II) those used as inlay sandwich technique. Clinical, radiographic and histological study were done to analyses the percentage of newly formed bone, the residual graft material, and marrow spaces/soft tissue.

### Pre-surgical preparation of patients: (for both groups)

Pre-operative clinical assessment of alveolar ridge and soft tissue coverage were done, in addition to assessment of occlusion and inter-arch distance.

**Fig. 1:**
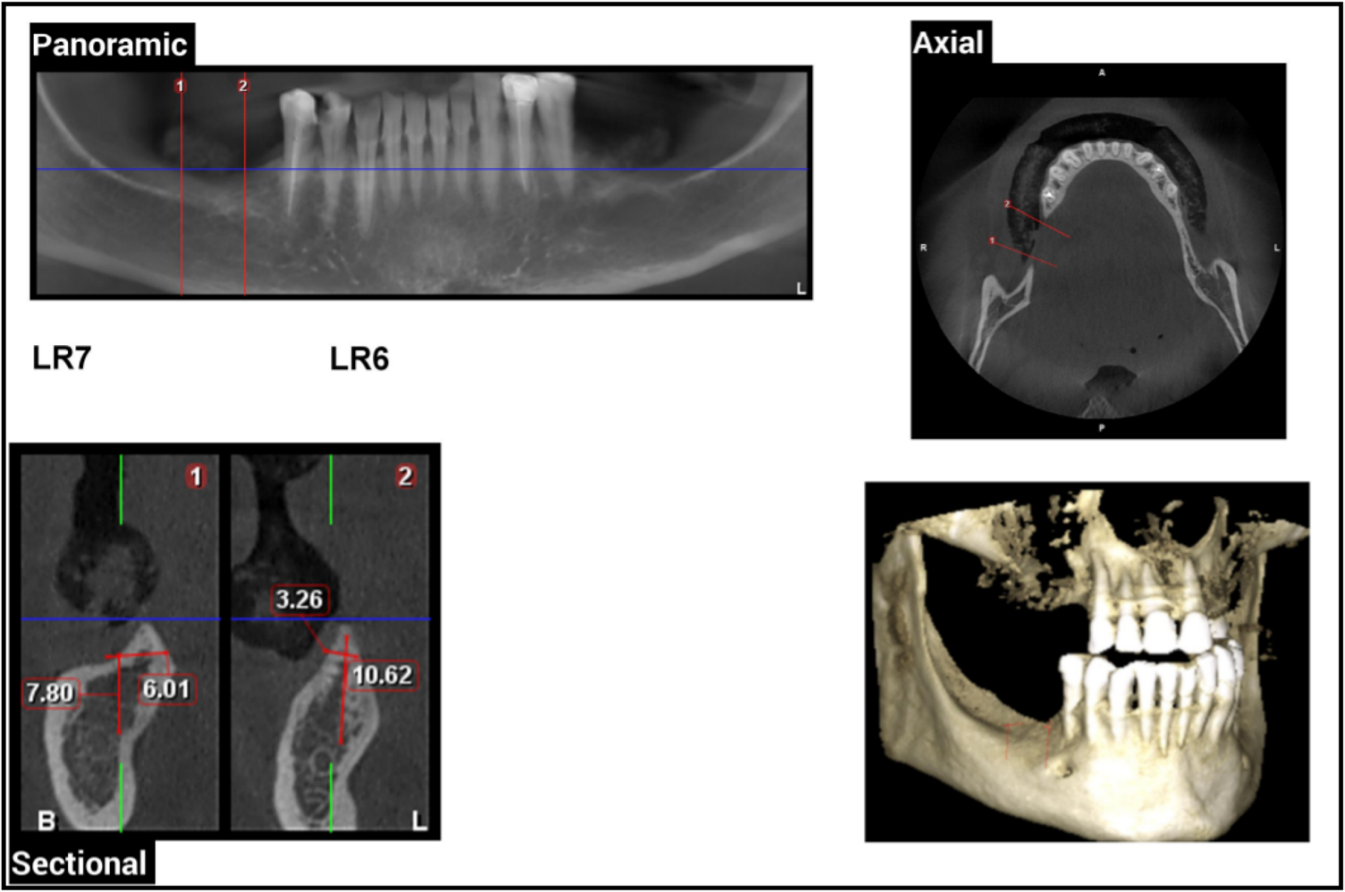
Pre-operative CBCT showing right mandibular posterior ridge deficiencies in Case Number 1 Group I, (1) LR7 Density = D3, (2) LR6 Density = D3

**Fig. 2:**
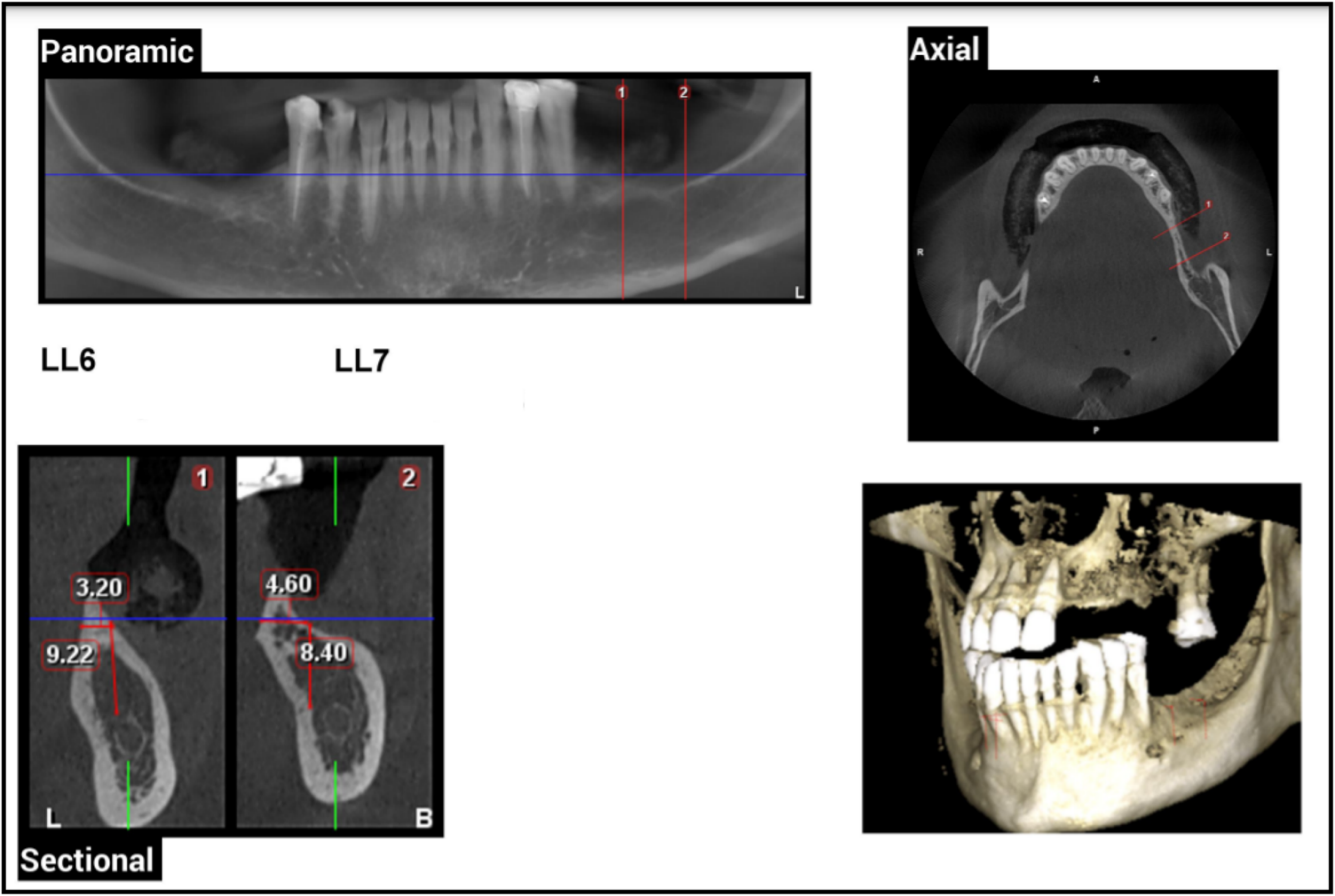
Pre-operative CBCT showing left mandibular posterior ridge deficiencies in Case Number 1 Group II, (1) LL6 Density = D3, (2) LL7 Density = D3

Pre-operative radiographic examination of the ridge height and relation of alveolar ridge crest to inferior alveolar canal which should ranging from 5 to 7 mm using CBCT (Figs. 1 and 2).

All patients were instructed proper oral hygiene instructions and underwent professional debridement one week before bone augmentation when necessary.

Envelopes containing the randomized codes were done to achieve randomization for the clinical study and were opened on the day of surgical procedure. Antimicrobial prophylaxis was obtained with the prescription of pre-operative antibiotic using 1 g of amoxicillin + clavulanic acid (Augmentin), starting one day before surgery and for the following 4 days. Corticosteroid (dexamethasone 8 mg, I.M) before surgical procedure and continued for the next 2 days every 12 h.

- Every patient was asked to rinse his mouth with 0.2% chlorhexidine HCL mouth wash for one minute just prior to surgery.
- Local anaesthesia used was Scandonest 2%.

## Augmentation procedure

### Group I: exposure of the recipient bed

All procedures were done under local anaesthesia. Incision was done using para-crestal incision through buccal mucosa respecting emergence of mental nerve, full thickness flap was retracted avoiding tension on mental nerve.

Exposure of recipient bed confirming bony architecture and size of bone block necessary for sufficient augmentation. The amount of bone needed was measured at the recipient site with a periodontal probe to outline the block to be harvested.

Preparation of the host site with perforation of the labial and crestal aspects of the host bone with a small diameter round bur. The holes were 3–5 mm apart through the entire area. Bone perforation was done under copious amounts of saline and penetrated both labial and crestal aspects of bone in the region of the graft bed.

### Exposure of the donor site

#### Harvesting of the bone block

Circum-Vestibular incision was made in the chin area at the bottom of the vestibule between mandibular canines through the mucosa 1–2 mm below the mucogingival junction followed by partial thickness dissection apically for 3 mm to preserve 3 mm of periosteum and mentalis muscle fibers on the bone, which will later be used to reattach the mentalis muscle.

**Fig. 3:**
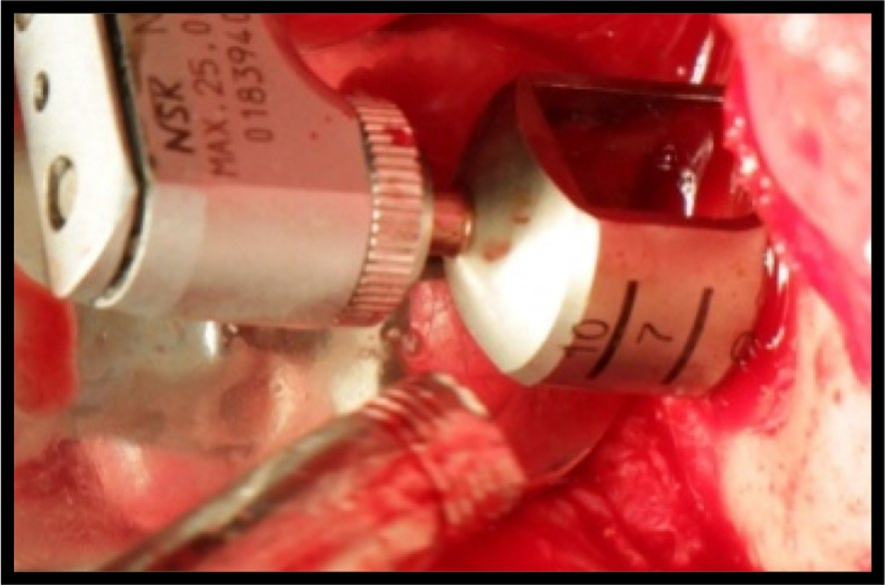
Intra-operative clinical photograph of the harvested chin graft using trephine bur in donor site of case number 1 Group I

The border of preplanned needed graft was cut and made to the depth of bone marrow using Trephine bur size 1 cm in Diameter to harvest the bone graft from the chin area (Fig. 3).

**Fig. 4:**
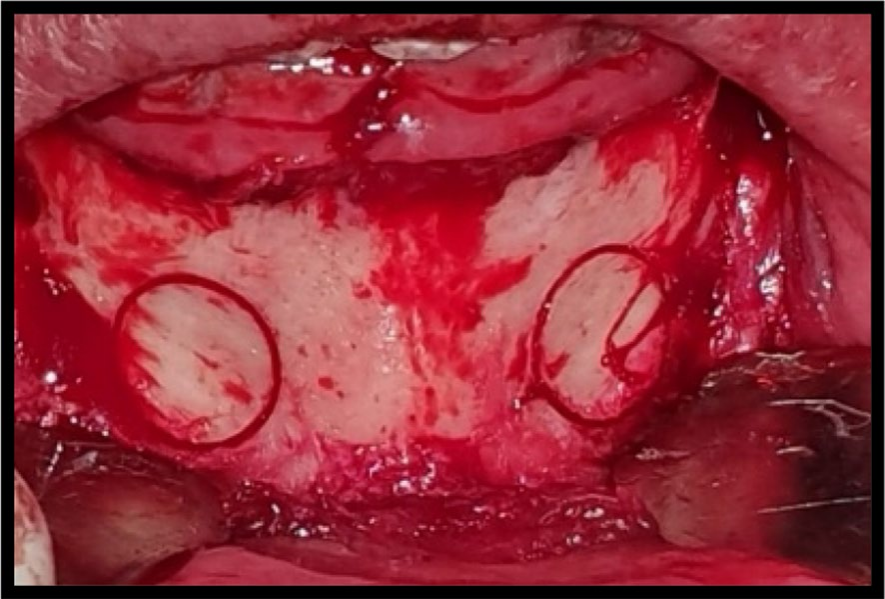
Intraoperative clinical photograph showing the harvested onlay chin graft in the donor site of case Number 1 Group I

When separating the cortical bone block from the marrow, (Fig. 4), the block was hold in place with a Kocher instrument while applying force to the osteotome to prevent loss of the specimen from the operating field.

Violation of the midline strut of bone in the most anterior portion of the symphysis, known as the mental protuberance, was avoided. When necessary two blocks can be harvested from each side of the midline, leaving a 3 mm midline strut to retain support for the chin profile.

After the block was removed, it was placed on a piece of gauze soaked with saline briefly while managing the bleeding that would be expected from the donor site. If necessary, bleeding can be controlled by insertion of a piece of gel foam.

**Fig. 5:**
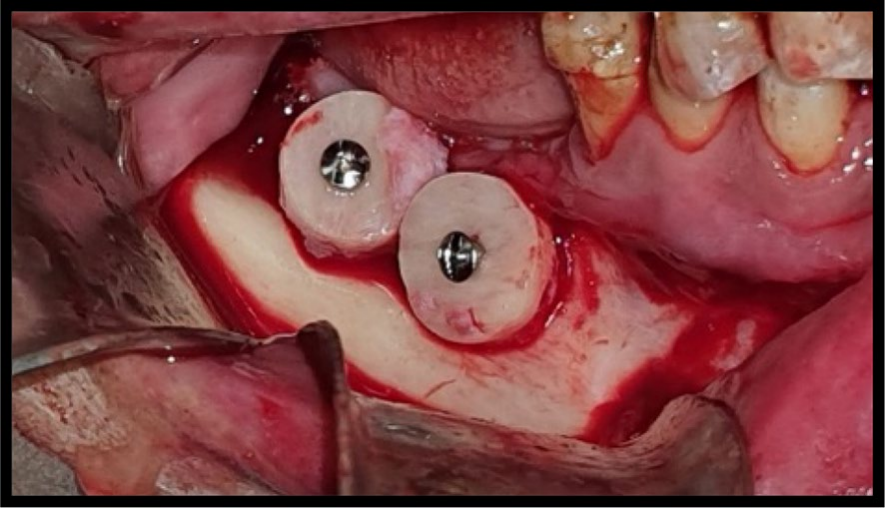
Intraoperative clinical photograph showing the harvested onlay chin graft fixated in the right mandibular defect of the recipient site with miniscrews of case Number 1 Group I

Block segment was then positioned over the recipient site with the endosteal side of the graft facing the fenestrated cortical bone. The block was trimmed conservatively and adapted to fit the defect site. To ensure immobility, the graft was fixed to the recipient site using titanium screws (le forte) (*D* = 2 mm *L* = 10–12 according to graft thickness) (Fig. 5).

### In group II: exposure of the recipient bed

Incision was done using para-crestal incision through buccal mucosa respecting emergence of mental nerve, full thickness flap was retracted avoiding tension on mental nerve, leaving the lingual mucosa attached to periosteum.

**Fig. 6:**
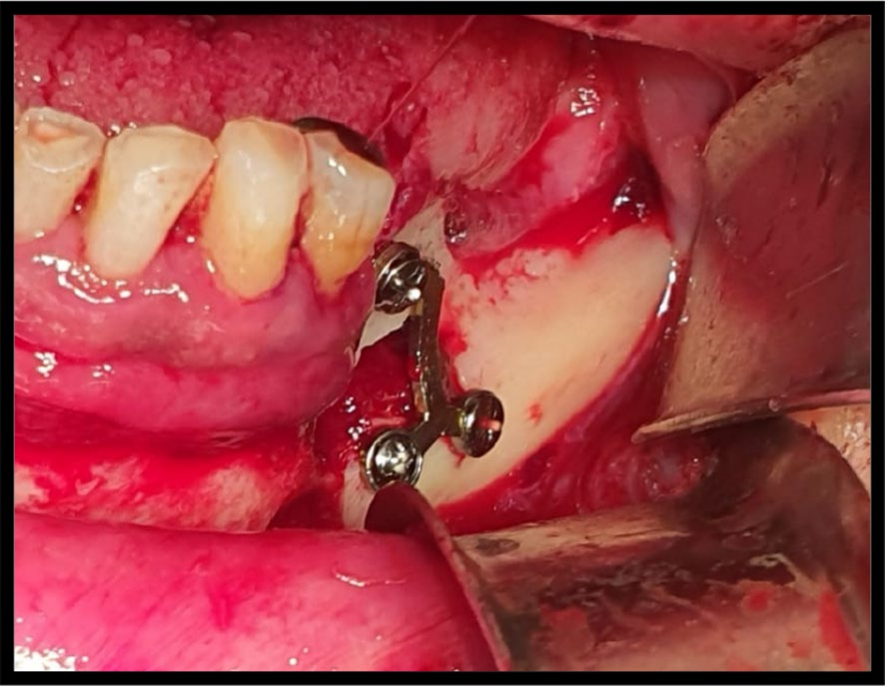
intraoperative clinical photograph showing the horizontal osteotomy of the left mandibular defect fixated with miniplate with 3 miniscrews in case Number 1 Group II

**Fig. 7:**
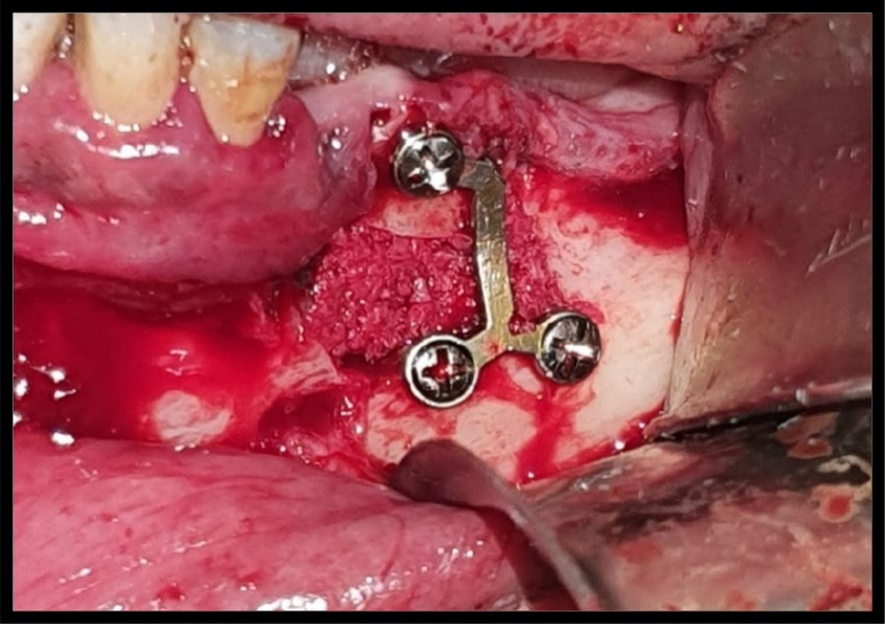
intraoperative clinical photograph showing the horizontal osteotomy of the left mandibular defect fixated with miniplate with 3 miniscrews with interpositional graft harvested with ACM bur from the symphysis area in case Number 1 Group II

Horizontal osteotomy was made at 3–5 mm from alveolar crest using saws. Two oblique cuts were made in coronal third of mandibular bone leaving at least 2 mm distal to last tooth in the arch. With the aid of the bone chisel, the osteotomies were completed. The osteotomized segment height was at least 3 mm. The segment was elevated preserving the lingual periosteum. Titanium miniplate with miniscrews were used to fix osteotomized crestal bone to basal bone (Figs. 6 and 7).

### Exposure of donor site

#### Harvesting of bone chips

Circum-Vestibular incision was made in the chin area at the bottom of the vestibule between mandibular canines through the mucosa 1–2 mm below the mucogingival junction followed by partial thickness dissection apically for 3 mm to preserve 3 mm of periosteum and mentalis muscle fibers on the bone, which will later be used to reattach the mentalis muscle.

Collecting bone with bone collector bur (ACM = Auto Chip Maker).

The harvested bone was inserted and adapted interpositionally in the pre-osteotomized fixated segments (Fig. 7).

##### Closure of both flaps

- Two layers wound closure of the donor site was done first followed by closure of the recipient site, using Vicryl (3/0) resorbable suturing material.
- At the recipient site, releasing incisions to the crestal incision of the flap was then made through the periosteum to allow flap advancement several millimeters.

Complete flap coverage and tension free wound closure was obtained.

Intra-oral pressure was applied by gauze pack which was removed 1 h post-operatively.

Pressure dressing (Chin bra) was placed for patients in both groups for at least 24 h, and a large harvest should have 3 days of pressure to reduce post-operative edema and ptosis of chin.

## Results

### Sample size calculation

Sample size calculated depending on a previous study. According to this study, the minimally accepted sample size was six per group, when the response within each subject group was normally distributed with standard deviation 2.7, the true mean difference was 5, when the power was 80% and type I error probability was 0.05.

### Statistical analysis

All data were presented as mean and standard deviation. Data were presented tables and graphs. Statistical analysis was performed with SPSS 16^®^ (Statistical Package for Scientific Studies), Graph pad prism and windows excel.

Exploration of the given data was performed using Shapiro–Wilk test and Kolmogorov–Smirnov test for normality which revealed that the significant level (*P* value) was insignificant as *P* value > 0.05 which indicated that data originated from normal distribution (parametric data) resembling normal Bell curve.

Accordingly, comparison between different groups was performed using independent *t* test, and comparison between different intervals was performed using Repetitive One-Way ANOVA test followed by Tukey’s Post Hoc test for multiple comparisons.

**Fig. 8:**
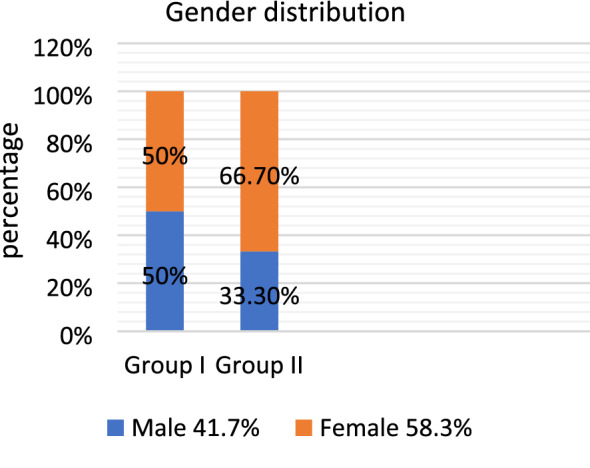
Bar chart representing mean % of gender distribution in both groups

- Demographic data
  1. Age: the age of patients in the BPF group ranged from 24 to 40 years with mean age 33.17 and standard deviation (SD) 6.4, which is closely to the L-PRF group as the age ranged from 21 to 42 years with mean age 32.67 and SD 7.6.
  2. Gender: the whole study included 7 female (58.3%) and 5 male (41.7%) patients. Group I included 3 female, 3 male patients, and Group II included 4 female and 2 male patients (Fig. 8, Table 1).

**Table 1:** Demographic data

Patient number	Age	Gender	Sites
Group I			
#1	40	Female	Right first, second molars
#2	38	Female	Left first, second molars
#3	40	Female	Right first, second molars
#4	24	Male	Left first, second molars
#5	29	Male	Left first, second molars
#6	35	Male	Right first, second molars
Group II			
#1	28	Male	Left first, second molars
#2	40	Female	Right first, second molars
#3	30	Female	Left first, second molars
#4	40	Female	Right first, second molars
#5	40	Female	Right first, second molars
#6	37	Male	Left first, second molars

### Follow up

Postoperative clinical and radiographic assessment were done along the follow up period.

- Clinical assessment

Postoperative clinical assessment included:

1. Signs of infection/wound dehiscence
2. Segment mobility
3. Pain
4. Infection/wound dehiscence and segment mobility

All patients in the present study were free from any signs of infection or wound dehiscence throughout all time intervals (D7, W2, W3 and W4) except for two patients in Group II have wound dehiscence with no graft failure (Table 2).

- Pain (VAS) visual analogue pain scale

**Table 2:** Showing wound dehiscence and segment mobility

		Group I	Group II
1	Wound dehiscence	0	2
2	Segment mobility	0	0

The pain scores for each patient in both groups was recorded immediate postoperatively and 1, 3 and 7 days postoperatively.

**Fig. 9:**
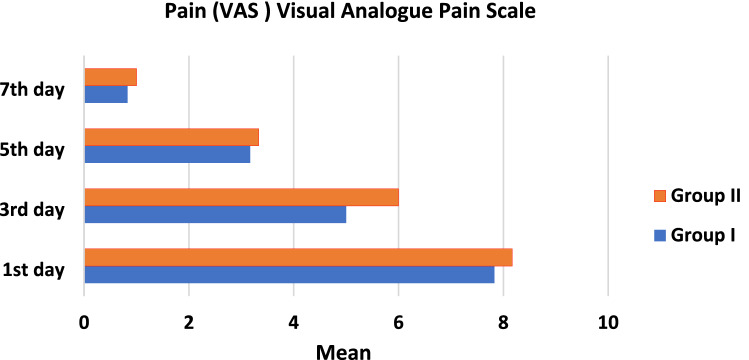
Line chart showing Mean of (VAS) Visual Analogue Pain Scale in both groups at different intervals

Mean and standard deviation of (VAS) of both groups at different intervals were presented in Table 3 and Fig. 9. Comparison between both groups revealed insignificant difference between them as *P* > 0.05, while comparison between different intervals revealed significant difference between them as *P* < 0.05 (there was a significant difference between each two intervals as all means have different superscript letters) in both groups.

**Table 3:** Mean and standard deviation of (VAS) Visual Analogue Pain Scale in both groups at different intervals and comparison between them

VAS	Group I		Group II		*P* value
	M	SD	M	SD	
1st day	7.83^a^	1.47	8.17^a^	1.17	0.66
3rd day	5.00^b^	0.89	6.00^b^	1.41	0.17
5th day	3.17^c^	1.17	3.33^c^	1.03	0.81
7th day	0.83^d^	0.75	1.00^d^	0.89	0.72
*P* value	< 0.0001*		< 0.0001*		

*P* probability level which is significant at *P* ≤ 0.05. Counts with the same superscript letters were insignificantly different as *P* > 0.05. Counts with different superscript letters were significantly different as *P* < 0.05

*M* mean, *SD* standard deviation

### Radiographic examination

Alveolar ridge height, this was assessed with CBCT at 1, 3 and 6 months postoperatively.

- Bone height

**Fig. 10:**
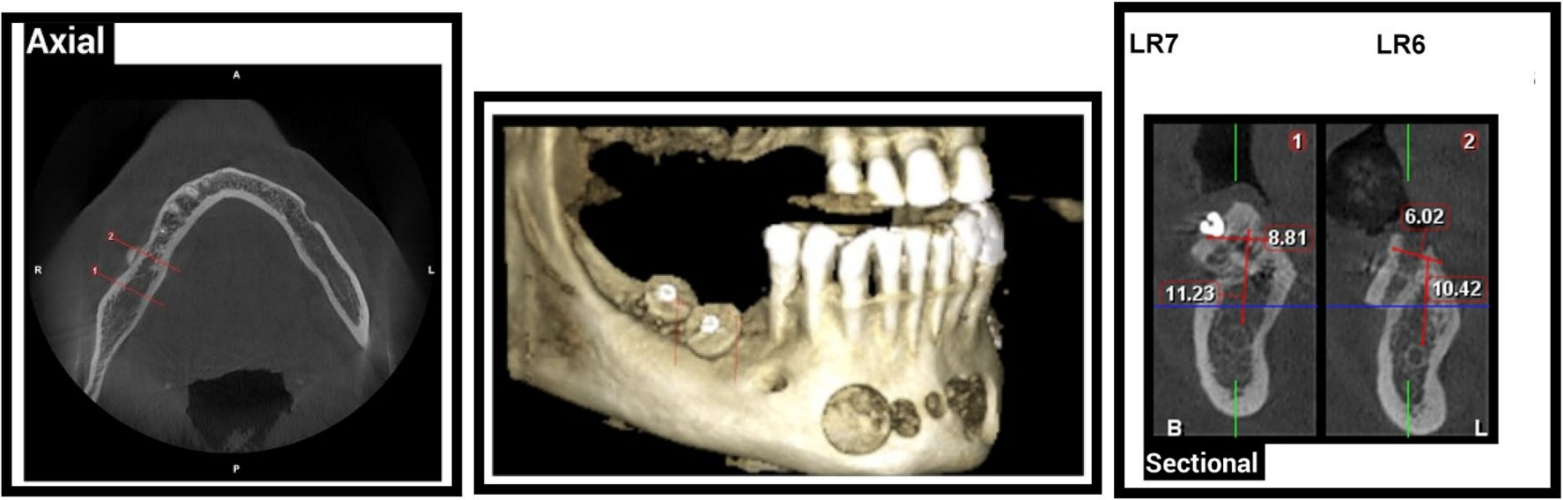
One-month post-operative CBCT showing right mandibular vertical augmentation using onlay chin graft in case Number 1 Group I, (1) LR7 Density = D3, (2) LR6 Density = D3

**Fig. 11:**
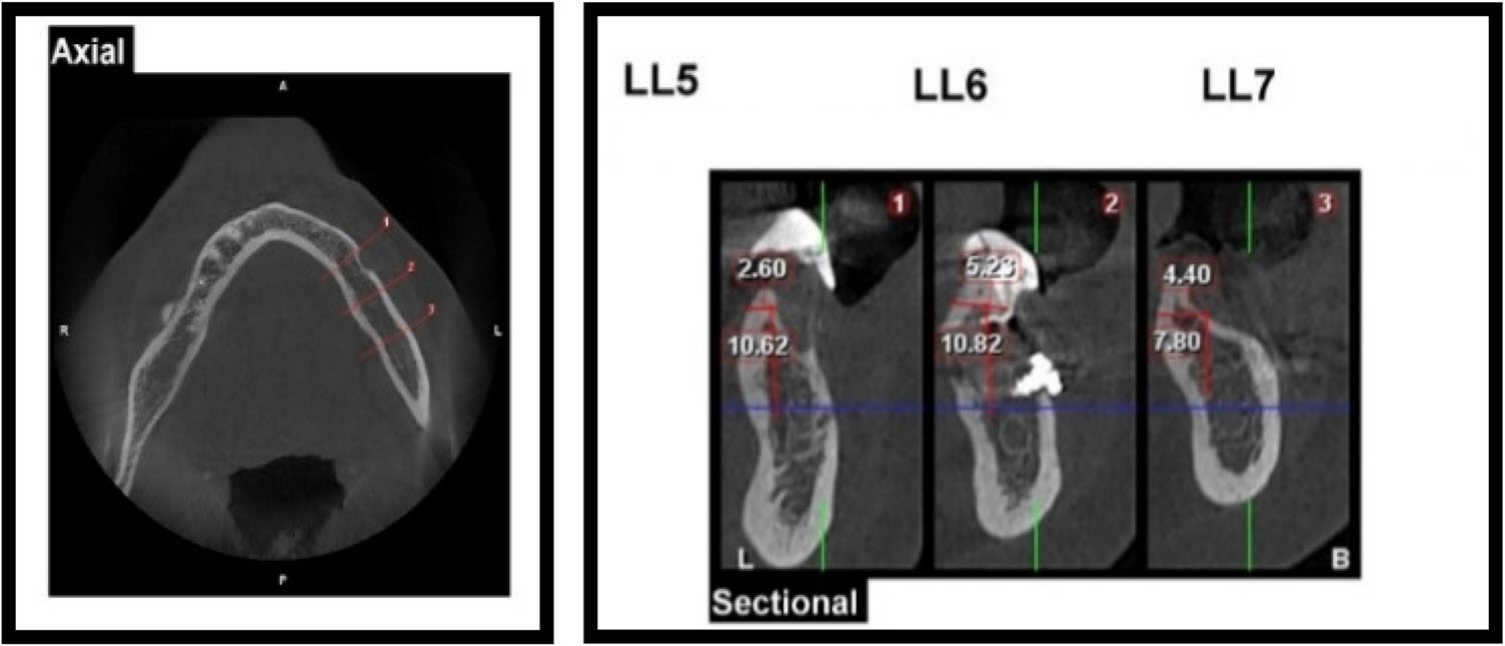
One-month post-operative CBCT showing left mandibular ridge augmentation using sandwich bone augmentation technique in case Number 1 Group II, (1) LL5 Density = D3, (2) LL6 Density = D3, (3) LL7 Density = D3

**Fig. 12:**
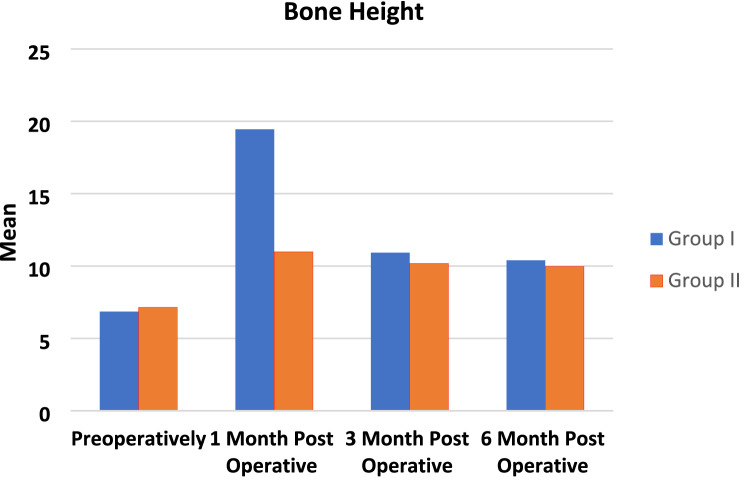
line chart showing Mean of bone Height in both groups at different intervals

Mean and standard deviation of bone height of both groups at different intervals were presented in Table 4 and Figs. (10, 11 and 12). Comparison between both groups revealed insignificant difference between them as *P* > 0.05, comparison between different intervals revealed insignificant difference between them as P > 0.05 in group I, while revealed significant difference in group II as *P* < 0.05 [Preoperatively was significantly the lowest (B), while there was insignificant difference between other intervals (A)].

**Table 4:** Mean and standard deviation of bone height in both groups at different intervals

	Group I		Group II		*P* value
	M	SD	M	SD	
Preoperatively	6.85^a^	1.06	7.14^a^	1.37	0.61
1 month post operative	19.45^a^	29.54	10.98^b^	0.74	0.49
3 month post operative	10.92^a^	0.99	10.18^b^	0.81	0.18
6 month post operative	10.39^a^	0.93	9.97^b^	0.51	0.35
*P* value	0.78		< 0.0001		
Percent of change	51.67%		39.6%		

*P* probability level which is significant at *P* ≤ 0.05. Counts with the same superscript letters were insignificantly different as *P* > 0.05. Counts with different superscript letters were significantly different as *P* < 0.05

*M* mean, *SD* standard deviation

Percent of change in bone height was also calculated and revealed that group I was higher than group II using the following formula.

$${\text{Percent}}\,\,{\text{change}}\,\, = \,\,\frac{{{\text{Final}} - {\text{Initial}}}}{{\left| {{\text{Initial}}} \right|}}\,\, \times \,\,100$$

After successful initial healing, chin grafts should be allowed to mature for 6 months prior to uncovering and implant placement. During the healing phase the block integrity was evaluated radiographically.

Reentry to the block site required re-incision of the overlying tissue, following the same incision lines used in the first surgery.

Block stability was assessed clinically by mucoperiosteal elevator to ensure immobility of the grafts. Plates and screws were removed.

## Discussion

Autogenous bone has been considered as the ‘gold standard’ for bone augmentation procedures due to its osteogenic, osteoinductive and osteoconductive properties, however, it suffers several disadvantages, such as higher morbidity, the need for a donor site, and the limited quantity of bone available [17]. Besides, autogenous bone block grafting presents a range of complications derived from the technique, occurring in 30–50% of cases [6, 9, 23]. Of these, the most serious is neurosensory disturbance, often observed in cases of autogenous chin bone harvesting, which can also produce aesthetic changes in the patient’s facial contours [5, 23].

The use of blocks or split blocks of autologous bone was considered one of the most common procedures for treating ridge defects. These augmentation techniques have shown an implant survival rate of 95–98% [3, 12]. Three studies [2, 13, 22] showed a 100% implant survival placed in regenerated areas after 1–3 years of follow-up, but there are studies with a shorter observation period and a smaller sample size. Nevertheless, these results are similar than those obtained by [16], with a 98.77% of survival rate of implants placed in bone regenerated with autogenous bone onlay blocks, compared to 82.8% of survival rate of implants placed in equine bone blocks [17].

In addition, in the systematic review of [18], a higher survival implant rate was obtained when autogenous bone blocks were used, in comparison to xenogeneic bone blocks.

Our study was in agreement with those done by [4] who stated that the combination of both characteristics in cortico-cancellous block grafts promotes early vascularization with maximum graft maintenance at the same time. It can be hypothesized that the difference between chin and ramus grafts due to its microarchitecture, since grafts from chin grafts have cortex lesser than the ramus graft with higher cancellous portion in comparison to those harvested from the ramus area [20, 25].

The decortication or the perforation of recipient site important for bone graft incorporation into host bone and even reduced graft resorption was observed compared to unperforated sites. The higher cortex bone density and lack of endosteal cells within the cortical bone that diminishes revascularization. The intimate dynamic interplay between angiogenesis and bone formation [15]. This observation was explained by faster vascularization, as shown by VEGF resulting in an accelerated bone remodelling process and increased bone apposition [7]. These perforations of the cortical bone provide a pathway for blood vessels and progenitor cells to approach the grafted compartment and has been routinely applied during GBR procedures [19]. Therefore the preparation of the recipient site may also contribute to the favourable results to our study.

## Conclusions

In conclusion, vertical ridge augmentation procedures using onlay chin graft took lesser time than the interpositional grafting with fixation technique, however, both techniques are promising for vertical ridge augmentation.

### Recommendation

We can decrease the surgical times using allograft or synthetic block graft instead of autogenous graft as interpositional graft in group II to minimize the surgical time.

## Data Availability

The data presented in this study are available on request from the corresponding author.
